# Recent Experimental Advances in Characterizing the Self-Assembly and Phase Behavior of Polypeptoids

**DOI:** 10.3390/ma16114175

**Published:** 2023-06-03

**Authors:** Liying Kang, Qi Wang, Lei Zhang, Hang Zou, Jun Gao, Kangmin Niu, Naisheng Jiang

**Affiliations:** School of Materials Science and Engineering, University of Science and Technology Beijing, Beijing 100083, China

**Keywords:** polypeptoids, self-assembly, crystallization, phase behavior, characterization methods

## Abstract

Polypeptoids are a family of synthetic peptidomimetic polymers featuring N-substituted polyglycine backbones with large chemical and structural diversity. Their synthetic accessibility, tunable property/functionality, and biological relevance make polypeptoids a promising platform for molecular biomimicry and various biotechnological applications. To gain insight into the relationship between the chemical structure, self-assembly behavior, and physicochemical properties of polypeptoids, many efforts have been made using thermal analysis, microscopy, scattering, and spectroscopic techniques. In this review, we summarize recent experimental investigations that have focused on the hierarchical self-assembly and phase behavior of polypeptoids in bulk, thin film, and solution states, highlighting the use of advanced characterization tools such as in situ microscopy and scattering techniques. These methods enable researchers to unravel multiscale structural features and assembly processes of polypeptoids over a wide range of length and time scales, thereby providing new insights into the structure–property relationship of these protein-mimetic materials.

## 1. Introduction

Polymers are capable of forming complex and organized structures that display multiple levels of hierarchy over a wide range of length scales. It is well-known that naturally occurring polymers, such as proteins, can be assembled into sophisticated multidimensional superstructures with hierarchical levels of structural arrangement, which are essential to their biological functionality, such as enzymatic catalysis, molecular recognition, and transport [1]. For synthetic polymers, a classic example is spherulite [2], a three-dimensional (3D) hierarchical structure that is often observed in semi-crystalline polymers such as polyethylene. Within a spherulite, polymer chains are packed and folded into ordered lamellar crystals at the molecular level, while they appear as stacked lamellae separated by amorphous regions with a sub-100 nm stacking periodicity. These mesoscale building blocks further aggregate into macroscopic spherulites that display a Maltese cross pattern under polarized light. 

The understanding and control of the hierarchical structure and the corresponding phase behavior of polymers have been pursued for decades, as they offer great opportunities for optimizing the macroscopic properties of polymeric materials, as well as creating new materials with novel functionalities. However, this is a challenging task due to the complex nature of polymer self-assembly or self-organization involved. Even for polyethylene, the simplest synthetic polymer that is semicrystalline, how the hierarchical structure formation is governed by nucleation and growth pathways has been discussed since the 1950s [3,4], and it is still under ongoing debate [5,6]. Unlike small organic molecules, the formation of hierarchically ordered structures by polymers is often dictated by a complex interplay between thermodynamic and kinetic driving forces, which involve short- and long-range non-covalent interactions (e.g., electrostatic, hydrogen bonding, π-stacking, van der Waals, and hydrophobic interactions), as well as entropic contributions, due to their long-chain nature. As chain relaxation and rearrangement can be easily impeded by these non-covalent interactions, hierarchical structures formed by self-assembly or self-organization are often trapped in various metastable states separated by energy barriers in the free energy landscape. This, in turn, allows polymers to create a multitude of non-equilibrium phases and hierarchical structures with diverse morphologies depending on their assembly pathways. While polymer characteristics such as chemical structure, monomer sequence, chirality, and chain architecture show significant impacts, external factors such as temperature, solvent, stress, and electric field can also influence the hierarchical structure formation and their corresponding phase behavior in many ways.

Inspired by nature, researchers have also been focused on developing protein-mimetic polymers that can achieve hierarchically ordered structures with a precisely controlled primary chain structure. These materials have great potential for biomedical and biotechnological applications, such as in drug delivery, biosensing, biomineralization, and tissue engineering [7]. Polypeptoids [8,9,10,11], a class of peptidomimetic polymers, are one of the examples of synthetic protein mimics. Unlike polypeptides, polypeptoids exhibit good thermal processability and solubility in various organic solvents, as well as enhanced protease stability, due to the absence of hydrogen bonding and stereogenic centers along the N-substituted polyglycine backbones [10,12,13]. There are two commonly used methods for accessing polypeptoids: solid-phase submonomer synthesis (SPSS) [14,15] and ring-opening polymerization (ROP) [16,17]. SPSS allows access to monodisperse sequence-defined polypeptoids, including oligopeptoids, with diverse side chain structures, which is advantageous for investigating hierarchical self-assembly with precisely controlled monomer sequences [10,18,19,20,21,22]. However, this method has limitations in efficiency of synthesis and is inaccessible for high-molecular-weight polypeptoids. On the other hand, ROPs of N-substituted glycine derived N-carboxyanhydride (R-NCA), or N-thiocarboxyanhydride (R-NTA) monomers using nucleophilic initiators (e.g., primary amines), can access well-defined polypeptoids, including block copolypeptoids, with high molecular weight and narrow dispersity due to their controlled/living nature [9,23,24,25]. Cyclic polypeptoids with tunable ring sizes can also be achieved by a controlled zwitterionic ROP mechanism [26,27,28]. With the synthetic advances of polypeptoids, a large variety of primary building blocks with tailorable chain length [29,30,31], side chain structure [32,33], chirality [10,34,35], sequence [15,20,36], and architecture [28,37] can be designed for assembling into hierarchical structures upon phase transition. 

In the last decade, researchers have reported a diverse range of hierarchical structures resulting from the self-assembly or self-organization of polypeptoids in solid and solution phases. These structures include low-dimensional nanostructures, such as nanofibers [29,38,39], nanotubes [40,41,42], nanosheets [30,33,43,44,45], as well as brush-like [46], sheaf-like [47], and flower-like superstructures [32], many of which have potential for a wide range of biomedical and biotechnological applications such as drug delivery, biosensing, antibacterial therapy, and gene therapy, etc. For example, the self-assembly of polypeptoids has been utilized to construct pH-sensitive vesicles or nanofibers [39], high surface area nanotubes [48], and highly crystalline fluoride nanoflowers [49] for drug delivery. These nanostructures offer enhanced drug loading capacity and improved drug delivery efficiency. To understand the fundamental relationships between the chemical structure, molecular arrangement, phase structure, and self-assembly/organization processes of these peptidomimetic materials, researchers have employed a range of characterization tools such as thermal analysis, microscopy, scattering, and spectroscopic techniques. Cryogenic transmission electron microscopy (cryo-TEM) [42,50], atomic force microscopy (AFM) [51,52], X-ray diffraction (XRD) [53,54], small-angle X-ray scattering (SAXS) [55,56,57], small-angle neutron scattering (SANS) [58,59,60], dynamic light scattering (DLS) [61,62], and circular dichroism (CD) spectroscopy [63,64] have all been useful in this regard. Scattering and microscopic techniques, that enable the in situ characterization of the structural evolution over a wide range of length and time scales under specific sample environments (e.g., at different temperatures, in solution settings, under various external stimuli, etc.), have been particularly helpful. In this review, we focus on some recent experimental advances in characterizing the hierarchical self-assembly and phase behavior of polypeptoids using, primarily, scattering and microscopic techniques. We use the term “polypeptoids” to refer to peptoid polymers of varying degrees of polymerization, including oligopeptoids, unless specifically mentioned otherwise. By introducing these characterization techniques, researchers have been able to access the multiscale structural features and evolution of structures during the assembly process of polypeptoids, leading to a better understanding of the formation mechanism of these protein-mimetic assemblies within the relevant length and time scales.

## 2. Recent Experimental Advances in Characterizing the Self-Assembly and Phase Behavior of Polypeptoids

Polypeptoids can undergo crystallization and self-assembly in various states, including bulk, thin film, and solution. In bulk state, polypeptoid chains are highly overlapped and form a condensed mass, which can be characterized using techniques such as differential scanning calorimetry (DSC), X-ray diffraction (XRD), and small-angle X-ray scattering (SAXS) to determine their multiscale structure and phase transition. The structural characterization of thin layers or films of polypeptoids that have been deposited on solid substrates often requires surface-sensitive techniques such as atomic force microscopy (AFM), X-ray reflectivity (XRR), and grazing incidence X-ray scattering or diffraction. In a dilute polymer solution, the concentration of polymer chains per unit volume drops significantly, leading to a limited amount of material available for analysis. Therefore, the in-depth structural characterization of a polymer solution typically requires advanced techniques such as cryogenic transmission electron microscopy (cryo-TEM), small-angle neutron scattering (SANS), and synchrotron-based solution X-ray scattering, which are often available only in shared instrumental facilities such as national laboratories. Nonetheless, by utilizing multiple characterization techniques and integrating the structural information obtained in different forms, researchers can gain a more comprehensive understanding of the self-assembly behavior of polypeptoids. In the following sections, we discuss recent experimental advancements in characterizing the self-assembly and phase behavior of polypeptoids in bulk, thin film, and solution states separately.

### 2.1. Polypeptoids in Bulk

Differential Scanning Calorimetry (DSC) is one of the most widely used thermal analysis techniques for studying the thermal behavior and phase transitions of polymeric materials. The principle of DSC is based on the measurement of the difference in heat flow between a sample and a reference material as they are subjected to controlled heating or cooling rates. During DSC measurements, the sample and reference materials are placed in separate pans, which are then heated or cooled at a constant rate while the temperature difference between them is monitored. The difference in heat flow between the sample and reference is recorded as a function of temperature or time to determine the change in enthalpy (∆H) or heat capacity associated with thermal transitions, such as glass transitions, melting, crystallization, and chemical reactions [65,66,67,68]. Depending on the specific instrument design, DSC can be divided into heat-flux type DSC, that measures the temperature difference, and power-compensation type DSC, that measures the electrical power or heat input required to keep the sample and the reference at the same temperature, where the “zero equilibrium” principle is realized [65,66,69,70,71]. Due to its rapid analysis, lack of special sample preparation requirements, and wide temperature range, DSC is widely used for preliminary studies on the thermal characteristics and phase transitions of newly synthesized polymers in many labs.

To gain a better understanding of the changes in structure associated with the phase transition of a polymer sample, DSC thermograms are often analyzed alongside results from scattering or microscopy measurements. Small- and wide-angle X-ray scattering (SAXS/WAXS) are commonly used techniques for determining the molecular packing, multiscale structure, and morphology of polymeric materials by measuring the intensities of X-rays that scatter from the sample [72,73]. The scattered intensity, I(*q*), is typically plotted as a function of the magnitude of the scattering vector *q*, which is described as *q =* 4πsin*θ/λ*, where *θ* is one half of the scattering angle and *λ* is the wavelength of incident X-rays. By using 2D area detectors, the scattering intensities, over a large range of *q*, can be collected simultaneously, depending on the sample-to-detector distance and the size of the detector. The observed that the 2D scattering pattern contains a wealth of structural information on the sample over a wide range of length scales, from a few angstroms to micrometers, relying on the electron density differences of the sample. Detailed data analysis is often achieved by integrating the 2D image to yield a 1D scattering profile, i.e., the I(*q*) versus *q* plot, where smaller *q* values correspond to larger observed length scales. Although laboratory-based X-ray sources can be used to perform SAXS/WAXS experiments, these often suffer from relatively low resolution and long data acquisition time, particularly for samples with a low volume fraction or concentration along the beam path. Alternatively, modern synchrotron X-ray sources with much higher brightness, flux, and beam coherence enable faster data collection and better data resolution, thereby allowing structural information to be resolved with a high level of precision even during in situ and in operando measurements.

**Figure 1:**
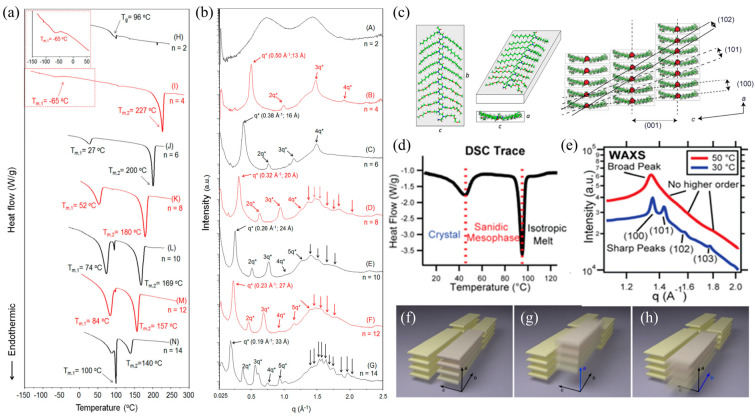
(**a**) DSC thermograms of linear poly (*N*-*n*-alkyl glycine) during the second heating cycle (*n* in the plot designates the number of carbons on the *n*-alkyl side chains); (**b**) WAXD diffractograms of cyclic poly(*N*-*n*-alkyl glycine)s at room temperature. (**c**) Relaxed molecular conformations of polypeptide backbones. Each molecule is a representative taken from a 288-molecule MD simulation and is shown from three angles. The parameters *a*, *b*, and *c* are the dimensions of peptoid molecules. (**d**) DSC trace of second heating process of poly(N-decylglycine)-block-poly(N-2-(2-(2-methoxyethoxy) ethylglycine)) with acetylated N-terminus (Ac-Ndc_9_-Nte_9_); (**e**) WAXS profiles at 50 °C (red line) and 30 °C (blue line) of Ac-Ndc_9_-Nte_9_; (**f**–**h**) Schematic illustrations of ordering in polypeptoid materials in (**f**) the crystalline phase and in (**g**,**h**) the sanidic liquid crystalline mesophase. Reprinted from Refs. [31,74,75] with permission from the American Chemical Society.

The structure and thermal behaviors of polypeptoids bearing various *N*-substituents have attracted attention since they became synthetically accessible. Lee et al. investigated the structure and thermal behavior of linear and cyclic polypeptoids with different alkyl side chains synthesized via ROP of R-NCAs [31]. It was found that poly(N-ethylglycine) (PNEG) is amorphous in nature, displaying only a glass transition during heating and cooling cycles (Figure 1a). X-ray diffraction (Figure 1b) showed two broad peaks or humps in the I(*q*) curves of PNEG, confirming its amorphous nature. Conversely, polypeptoids bearing *n*-alkyl side chains longer than two carbons are crystallizable and display well-defined principal diffraction peaks (q*) and multiple higher order reflections (2q*, 3q*, 4q*, and 5q*) (Figure 1b), indicating the formation of lamellar-like crystalline structures at room temperature. These samples also exhibit two first-order endothermic transition temperatures in the DSC thermograms during the heating cycle, i.e., *T*_m,1_ (∆H_1_) and *T*_m,2_ (∆H_2_) (Figure 1a), as well as two first-order exothermic peaks upon cooling from the melt. Notably, these first-order phase transitions are strongly coupled; upon increasing side chain length, there is a systematic increase in *T*_m,1_ (∆H_1_) and decrease in *T*_m,2_ (∆H_2_). This indicates that increasing the side chain length promotes side chain crystallization while weakening the main chain crystallization of polypeptoids.

The packing motif of polypeptoids bearing n-alkyl side chains was further elucidated by Greer et al. using X-ray diffraction and molecular dynamics simulations [74]. They found that polypeptoids adopt board-like molecular conformations in the crystalline phase, where the *cis*-amide backbone is fully extended and approximately coplanar with the *n*-alkyl side chains (Figure 1c). By examining the lattice dimensions of various polypeptoid samples obtained by X-ray scattering, they revealed the universal relationship between molecular parameters (i.e., numbers of backbone-repeating units and carbons on the *n*-alkyl side chains) and the unit cell dimensions of polypeptoids in the crystalline phase. Using in situ high-temperature X-ray scattering, they later evidenced that the lower-temperature phase transition corresponds to a crystalline phase to a “sanidic” liquid crystalline (LC) mesophase transition, while the higher-temperature transition corresponds to the LC mesophase to isotropic melt transition (Figure 1d) [75]. Heating above *T*_m,1_ results in a loss of long-range correlation between face-to-face and side-by-side packings in the “sanidic” LC mesophase, as indicated by the broadening of the (100) peak and disappearance of higher-order reflections (101), (102), and (103) (Figure 1e–h). In addition, the thermal behavior of polypeptoids is mainly affected by the *N*-terminal group; all polypeptoids with an acetylated *N*-terminal exhibit two thermal transitions, whereas polypeptoids with unacetylated *N*-terminal show only one transition between sanidic LC mesophase and isotropic melt [75]. 

### 2.2. Polypeptoid Thin Films and Monolayers

In addition to bulk samples, ultrathin films and monolayers of polypeptoids prepared on flat solid substrates offer a unique opportunity to investigate their crystallization and phase behavior. Application-wise, thin layers of protein-mimetic also hold promise for applications in antifouling coatings, biosensors, and bioelectronics. It is well known that, when the film thickness is comparable to the size of the unperturbed polymer chain, geometric confinement impedes the isotropic growth of polymer crystals into complex three-dimensional structures [76], such as spherulites. Instead, polymer crystals often adopt distinct quasi-two-dimensional morphologies under nanoconfinement with specific molecular orientations with respect to the substrate [5,77]. 

Atomic force microscopy (AFM) is a powerful tool for investigating the crystallization and self-assembly of nanoconfined polymer thin films. It uses a very sharp tip (usually <10 nm in diameter) attached to a flexible cantilever as the force sensor to scan over the surface of a sample, recording the interaction between the tip and the sample to acquire images in real space. Compared to other microscopic techniques, such as optical microscopy (OM) and transmission electron microscopy (TEM), AFM has several advantages in the study of polymer crystallization and self-assembly, including high spatial resolution (sub-nanometer laterally and sub-angstrom vertically), easy sample preparation, and the ability to measure under ambient conditions. In situ AFM measurements at different temperatures or in solution can be performed by using temperature-controlled sample stages or liquid cells. In reciprocal space, grazing incidence X-ray scattering (GIXS) or diffraction (GID) is often used in conjunction with AFM to probe the molecular packing and multiscale structural ordering of polymer thin films. In this approach, a high-quality monochromatic X-ray beam is directed at a very low angle onto the surface of a thin film, allowing for the detailed study of the polymer surface and interfaces [78,79,80,81]. By controlling the incident angle of X-rays, it is possible to selectively probe the surface and interior regions of a film, offering valuable information on the molecular ordering, chain orientation, and microstructure formation of the polymers as a function of distance from the substrate interface [82,83]. To achieve accurate structural information, it is crucial that the sample for GIXS or GID measurements is flat and free of surface defects. Additionally, the sample substrate should be stable, has low X-ray absorption, and contributes minimally to the scattering signal. 

**Figure 2:**
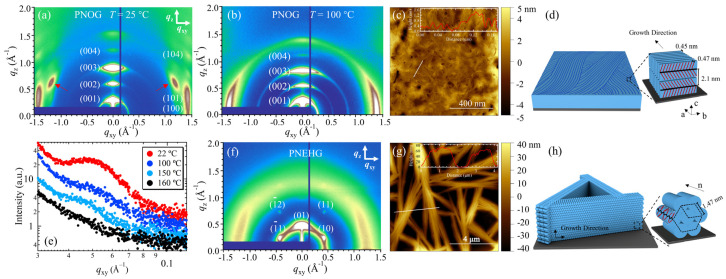
These are 2D GIWAXD images of the 50 nm thick PNOG films measured at (**a**) RT (T < T_C_) and (**b**) 100 °C (T_C_ < T < T_LC_), respectively. The out-of-plane (q_z_) and in-plane (q_xy_) directions are indicated by arrows. (**c**) Representative AFM height image of the PNOG films recrystallized from isotropic melt via slow cooling; the cross-sectional height profiles along the white lines are shown in the inset. (**d**) Schematic illustrations of the molecular packing and hierarchical crystalline structure of PNOG thin films on solid substrates. (**e**) In situ high-temperature 1D GISAXS profiles for the PNOG films along q_xy_ during the cooling process. The corresponding 2D GIWAXD images measured at RT, AFM height image, and schematic illustrations of the molecular packing and hierarchical crystalline structure of PNEHG film are shown in (**f**–**h**), respectively. Reprinted from Ref. [47] with permission from the American Chemical Society.

In a recent study by Wang et al., in situ high-temperature grazing incidence X-ray diffraction (GIWAXD) was used in conjunction with atomic force microscopy (AFM) to investigate the melt recrystallization behavior of nanoconfined polypeptoid films prepared on silicon substrates [47]. Two polypeptoids, poly(*N*-octyl glycine) (PNOG) and poly(*N*-2-ethyl-1-hexyl glycine) (PNEHG), were used to prepare thin films to investigate the effect of side chain branching. In the molten state (*T* > *T*_m_), both PNOG and PNEHG are fully disordered with randomly oriented chains, as observed in the amorphous halo rings in the 2D GIWAXD patterns. Interestingly, after being slowly cooled to room temperature from isotropic melt, the recrystallized PNOG film at 25 °C shows a series of (00*l*) reflections in the out-of-plane (*q*_z_) direction (Figure 2a), corresponding to the side-by-side molecular packing of board-like PNOG that is separated by *n*-octyl side chains in the substrate-normal direction (Figure 2c). The well-defined (10*l*) reflections also indicate a long-range correlation between face-to-face and side-by-side packings in the crystalline phase [74,75]. The near-perfect orthorhombic crystal structure of PNOG at the molecular level further leads to the formation of long-range ordered worm-like lamellar crystals that occupy the entire film with an average width of ~13 nm, as evidenced by GISAXS and AFM (Figure 2c–e). Interestingly, in the “sanidic” LC mesophase, the 2D GIWAXD pattern collected at 100 °C showed a lack of long-range correlation between face-to-face and side-by-side packing, as evidenced by the absence of higher-order reflections, despite the majority of molecules still being oriented edge-on (Figure 2b). The absence of off-axis peaks in GIWAXS indicates that the *n*-octyl side chains are disordered in the LC mesophase. In addition, in situ high-temperature X-ray scattering allowed for the correlation between the molecular packing of PNOG and the formation of worm-like lamellar crystals to be revealed during the cooling process.

In contrast to PNOG, it was found that the PNEHG film displays a typical diffraction pattern that corresponds to a hexagonal lattice when the melt recrystallized (Figure 2f). The asymmetric branching of the 2-ethyl-hexyl side chains results in a rod-like molecular geometry with significant steric hindrance, causing the side chains to splay out along the extended backbone. This geometry drives PNEHG molecules to stack into a columnar hexagonal (Col_hex_) mesophase, with their backbone or the molecular director (*n*) aligned parallel to the substrate due to the confinement effect (Figure 2h). At larger scales, PNEHG molecules assemble into hierarchically ordered, sub-micrometer-thick sheaf-like superstructures consisting of stacked fibrous lamellae (Figure 2g,h). In contrast to the densely packed worm-like PNOG lamellae, the PNEHG lamellae grew to a height that was approximately 30% greater than the initial thickness of the as-cast film. This growth resulted in the consumption of amorphous materials and the formation of microscopic holes in the film (Figure 2g,h).

### 2.3. Polypeptoid Solutions

Solution self-assembly of amphiphilic block copolymers (BCPs) is a widely used method to create well-defined nano-/meso-structures with tailorable size and functionalities. For amphiphilic BCPs comprising non-crystallizable blocks, the commonly observed morphologies include spherical micelles, worm-like micelles, and vesicles with a core-shell type of architecture in a selective solvent [84]. On the other hand, by using amphiphilic BCPs that consist of at least one crystallizable block as the primary building block, one can create core-crystalline micelles that display highly anisotropic morphologies via the so-called crystallization-driven self-assembly (CDSA) approach [85,86]. Examples include one-dimensional (1D) nanorods [87,88], two-dimensional (2D) nanosheets or platelets [89,90], as well as more sophisticated hierarchical assemblies [91]. These crystalline nanostructures can be sterically stabilized against aggregation or precipitation in the solution by introducing a solvophilic block. In some cases, CDSA can proceed in a “living” fashion, enabling precise control over the size and dimension of these BCP nanostructures [92,93,94,95]. 

Over the past decade, solution self-assembly, as well as CDSA of peptoid-based BCPs, have also been extensively studied [29,30,32,33,96]. Since the final micellar morphology created by CDSA highly relies on the assembly pathways, which can be greatly influenced by sample preparation/processing conditions [97,98], it is crucial to monitor the process of BCP self-assembly in solution. In this regard, various microscopic and scattering techniques, such as AFM, TEM/cryo-TEM, and light/X-ray/neutron scattering, have been used to characterize not only the final solution morphology of peptoid-based BCPs but also the structural changes as a function of time or temperature, providing a comprehensive understanding of the assembly mechanism.

**Figure 3:**
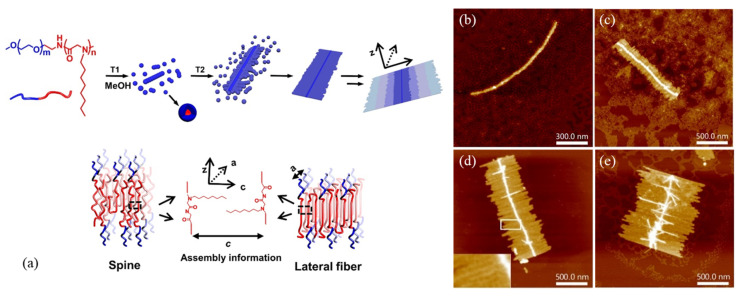
(**a**) Superbrush structures and molecular arrangement of both spine and lateral fibers of PEG_112_-*b*-PNOG_54_; AFM images of PEG_112_-*b*-PNOG_54_ (**b**) annealing at 65 °C for 2 h in 0.5 mg/mL methanol solution, and incubated for (**c**) 1.5 h, (**d**) 3 h, and (**e**) 7 h. Reprinted from Ref. [46] with permission from the National Academy of Science.

AFM is a widely used technique for tracking the morphological evolution of polypeptoids during solution self-assembly. By depositing the sample, at a given time or temperature during solution self-assembly, onto Si or mica substrate and evaporating the solvent immediately, it is possible to “freeze” the self-assembled nanostructures or morphology from solution. The dilution of the sample solution is often required to isolate the crystals or micelles from aggregation during solvent evaporation. However, it is sometimes difficult to ensure that the observed AFM images in the dry state truly reflect the actual solution morphology. Thus, it is recommended to interpret the AFM results carefully in conjunction with other characterization methods. In recent years, several research groups have used AFM to investigate the CDSA behavior of peptoid-based BCPs in dilute solution, including the formation of nanofibers, nanosheets, brush-like, and flower-like superstructures [29,30,32,46]. For example, Sun et al. [46] used AFM to study the hierarchical assembly process of planar nanobrush structures from spherical micelles of poly(ethylene glycol)-*block*-poly(N-octyl glycine) (PEG-*b*-PNOG) in methanol (Figure 3a). It was found that the nanobrush formation of PEG-*b*-PNOG can be controlled in a “living” fashion by tuning the interplay between crystallization and solvophobicity. At an elevated temperature in the LC mesophase of PNOG, the initial spherical micelles first assemble into long necklace-like spines (Figure 3b). Upon cooling to a lower temperature, where the driving force for crystallization becomes dominant, the long spine serves as seed crystals that allow the further fusion of micelles into short lateral fibers on the side surface of the spine (Figure 3c,d). The effect of micelle concentration, annealing conditions, and solvent on the solution morphology and self-assembly pathway was also systematically studied by AFM along with other techniques.

In addition to regular AFM measurements performed under ambient conditions, in situ AFM using a liquid cell allows the monitoring of assembly pathways of polymeric systems in liquid environments. By collecting in situ AFM images at different time intervals, the structural evolution and assembly kinetics of polymeric nanostructures in a solution or near a solution–substrate interface can be directly observed. For example, Jiao et al. [22] investigated the hierarchical self-assembly of β-cyclodextrin (CD)-modified sheet-forming peptoids (Pep-cd) using in situ AFM images collected over time in aqueous solutions under acidic conditions on different substrates. The self-assembly involves the formation of small spheroidal precursors, which subsequently assemble into 2D membranes or 3D intertwined ribbons, depending on the solution and substrate conditions. The assembly mechanisms of these hierarchical structures, arising from distinct interactions between Pep-cd segments and solid substrates in different solutions, were discussed, with in situ AFM providing direct experimental evidence.

Transmission electron microscopy (TEM) is a widely used tool for characterizing the crystalline structure and morphology of polymer assemblies with nanometer-scale resolution. By utilizing a beam of energetic electrons that passes through an ultrathin specimen, it is possible to visualize the morphological features in a bright field image, even without staining the sample. In reciprocal space, the crystalline structure and unit cell dimensions of the polymer assemblies can also be obtained via electron diffraction. However, conventional TEM imaging suffers from limited spatial resolution, low image contrast, electron beam damage, and structural changes during sample preparation. When studying the solution self-assembly of polymeric materials, removing the sample from its native solution environment can cause structural changes, potentially leading to an inaccurate interpretation of the self-assembly behavior. To address this challenge, liquid phase TEM (LP-TEM) can be used, utilizing a liquid cell instead of a vacuum-transfer specimen holder. However, LP-TEM often suffers from radiation damage to the sample caused by the interaction of the energetic electrons with polymeric samples. In contrast, cryo-TEM offers an alternative approach that preserves the native state of unstained samples by rapidly freezing them and reduces radiation damage by imaging them at liquid nitrogen temperature using a low electron dose [99]. Moreover, machine-learning-based image processing algorithms can be utilized to reconstruct high-resolution 3D structures of the assembly from numerous noisy cryo-TEM micrographs [100]. 

**Figure 4:**
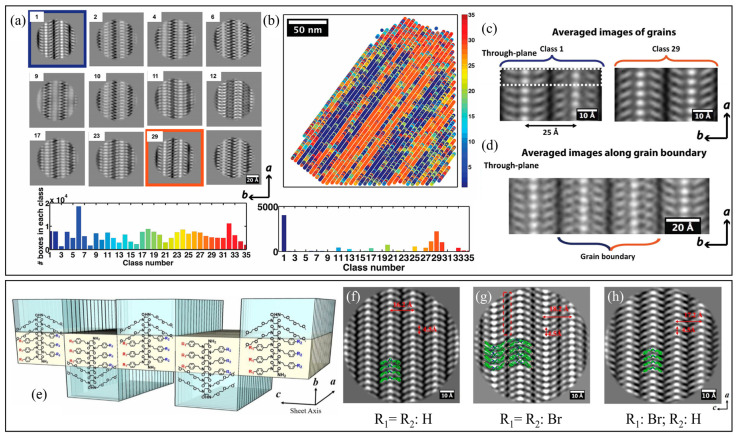
Structural variations of Nte-*b*-Npe crystals and distribution map. (**a**) A total of 12 dominant classes were founded from 35 classes. (**b**) Distribution of classes in one micrograph. The two major classes, 1 and 29, are mirror images of each other. Different colors correspond to the average values of 35 different classes. (**c**) Averaged images obtained by sorting unit cells. The most highly populated classes are classes 1 and 29. (**d**) Image obtained by averaging boxes along the domain boundaries. Reprinted from Ref. [101] with permission from the American Chemical Society. (**e**) Schematic illustrating nanosheet structures of polypeptoids; molecular chains are packed antiparallel along the *c* direction and parallel along the *a* direction. The hydrophobic block (yellow color) is crystalline and the hydrophilic block (blue color) is amorphous. Cryo-TEM of (**f**) Nte_4_-Npe_6_, (**g**) Nte_4_-N4Brpe_6_, and (**h**) Nte_4_-(N4BrpeNpe)_3_ from *b* direction (top view). Reprinted from Ref. [104] with permission from the National Academy of Sciences.

Using low-dose cryo-TEM, Jiang et al. [101] investigated the crystal packing of free-floating peptoid nanosheets that self-assembled from amphiphilic Ac-pNdc-*b*-pNte, i.e., poly(N-decylglycine)-*block*-poly(N-2-(2-(2-methoxyethoxy) ethylglycine)) with acetylated N-terminus, in water. To achieve the atomic-scale imaging of peptoid nanosheets, the authors applied single particle analysis [102,103], a cryo-EM analytical method commonly used by structural biologists for the 3D reconstruction of protein molecules. The electron micrographs were first divided into small boxes composed of unit cells, which were then classified and averaged to reveal the shape and positioning of individual chains and to map out the distribution of structural heterogeneity in the nanosheet (Figure 4a,b) [101]. By using sorting and averaging algorithms, they achieved the high-resolution imaging of synthetic polymer crystals using cryo-TEM and, for the first time, visualized the crystalline grains and grain boundaries of unstained peptoid nanosheets with an atomic level (∼2 Å) resolution (Figure 4c,d). To further investigate the effect of side-chain chemistry on the structure of crystalline nanosheets, Xuan et al. [104] later synthesized a series of diblock copoly-peptoids (Nte-b-Npe) that consist of a hydrophilic pNte block and different N-2-phenylethylglycine (Npe)-based hydrophobic blocks with various aromatic ring substituents, including H, methyl, and halogen atoms such as Br and I (Figure 4e). Low-dose cryo-TEM imaging, along with single-particle analysis, was used to study the self-assembly of Nte-*b*-Npe in an aqueous/tetrahydrofuran solution. The Nte-*b*-Npe 2D nanosheets displayed the same *cis* backbone fold in the crystallographic *b* direction, but the crystal lattices adopted different packing geometries along the *c* direction, depending on the para substitution of the aromatic side chains (Figure 4f–h). For an asymmetric sequence with alternating phenylethyl and 4-bromophenylethyl side chains, the sheets exhibited all parallel V-shapes, and the side chains with different lengths on each side were noticeable (Figure 4h). 

**Figure 5:**
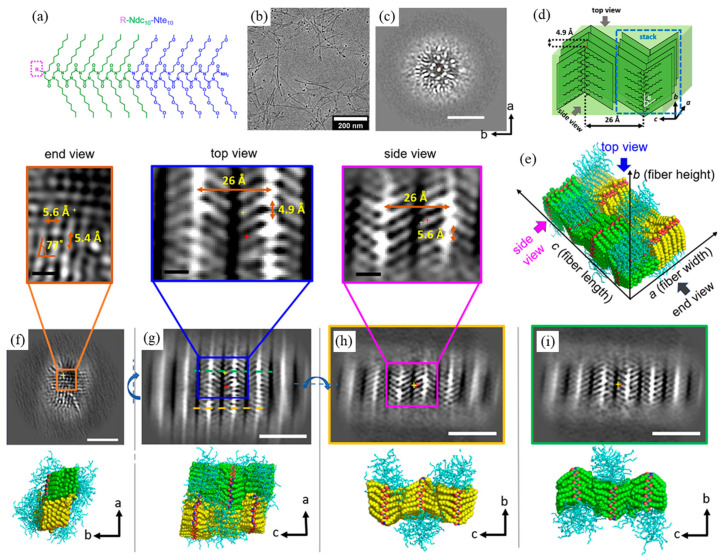
(**a**) Molecular structure of R-Ndc_10_-Nte_10_. (**b**) Low-dose cryo-TEM micrographs of H-Ndc_10_-Nte_10_ in water. (**c**) 3D cryo-TEM reconstruction of H-Ndc_10_-Nte_10_ nanofibrils in water in *ab* cross section. (**d**) Schematic of the molecular arrangement of Ndc chains in the crystalline lattice. (**e**) Molecular representation of peptoid packing geometry in the H-Ndc_10_-Nte_10_ nanofibrils in urea. Slices from the cryo-TEM 3D reconstructions from the (**f**) end view (*ab* cross section), (**g**) top view (*ac* cross section), and (**h**,**i**) side views (*bc* cross sections) of the nanofibrils. Scale bar is 5 nm in (**b**,**c**,**f**–**i**) and 10 Å in the enlarged images. Note that the yellow + represents the position of the slice, and the red + represents the center of the fibril model. The blue curved arrows in (**f**–**h**) represent the rotation directions. The black thin arrows in (**c**–**i**) represent the directions of crystallographic axes. Reprinted from Ref. [105] with permission from the American Chemical Society.

Recently, Yu et al. [105] used cryo-TEM 3D reconstruction to directly visualize the conformation of an individual poly(*N*-decylglycine)-*block*-poly(*N*-2-(2-(2-methoxyethoxy)ethoxy)ethylglycine) (Ndc_10_-*b*-Nte_10_) within a peptoid nanofiber (Figure 5a,b) in real space. All the end- (*ab* plane), top- (*ac* plane), and side-view (*bc* plane) cross-sections were identified and analyzed in the reconstructed 3D model (Figure 5d,e). In the end-view cross-section image (Figure 5c), they found that the nanofibrils comprise a Ndc_10_ hydrophobic crystalline core surrounded by a less bright outer layer consisting of amorphous hydrophilic Nte_10_ blocks (Figure 5c). In the top-view image (Figure 5g), similar to a previous work by Xuan et al. [104], the molecular stacks in the nanofiber lattice also exhibited V-shaped arms with the side chains extending out on both sides of the backbones along the *c* direction. Notably, the individual peptoid chain in the side-view cross-section was, for the first time, directly observed using cryo-TEM (Figure 5h,i). Furthermore, based on the side-view cross-sections (Figure 5h,i), it was evident that the arms in the same column were angled in opposite directions, indicating that the two segments have inverted symmetry along the *a* direction. The spacing between the side chain methyl groups along the *a* direction and the *c* direction was 4.9 ± 0.2 Å and 26 ± 0.5 Å (Figure 5g), respectively. In addition, SPA 3D reconstruction results showed that the distance between adjacent repeating side chains along the backbone (*b* direction) is 5.6 ± 0.1 Å with a tilt of 77° (Figure 5f), which is consistent with the data in the *ab* plane (Figure 5h). This suggests that the peptoid molecules adopted an extended, all-*cis*-sigma strand conformation in the crystal lattice, in good agreement with the molecular picture previously suggested [29,74,75]. They also demonstrated that the presence of exogenous small molecules (e.g., urea, formamide) can greatly enhance the long-range ordering of peptoid molecules along the *a* direction, highlighting the significant role of the chain terminus in molecular packing.

While real-space microscopy imaging can provide local structural information with atomic-scale resolution, scattering techniques using X-ray or neutron sources can provide averaged structural information over a large sample volume in reciprocal space, in situ and in solution environments. However, despite sharing the same principle as the typical transmission X-ray scattering measurement described in Section 2.1, solution SAXS/WAXS requires additional steps and precautions in order to perform a reliable quantitative analysis. For synchrotron-based solution scattering, a single-sample measurement is often divided into multiple short-time exposures with pauses of at least a few seconds in between exposures to reduce radiation damage to polymeric samples. Such measurements are often accompanied by the use of a flow cell device that allows the constant movement of the sample solution within the capillary tube. If unexpected continuous changes in the scattering profile occur among different exposures, it is likely that radiation damage has occurred [106]. Otherwise, these multiple frames, which provide similar scattering profiles, can be averaged to improve the signal-to-noise ratio. Another important aspect is to subtract the scattering contributions from the solvent background and empty cell from the total solution scattering to obtain the scattering contribution of the polymers alone. This requires additional exposures from the pure solvent by using the same cell with the same X-ray path length through the sample. For polymeric micelles or assembled nanoparticles, the SAXS region contains information regarding the size and shape of individual polymer particles, as well as the spatial correlation among these particles, while the WAXS region reveals the molecular packing and conformation of the polymer chains within the nanoparticles at smaller length scales [107]. When the solution has a dilute particle concentration, the structure factor becomes negligible, and the background-subtracted SAXS curve represents only form factor contributions. This can benefit researchers who are interested in the detailed structural features of individual self-assembled nanoparticles. However, solution self-assembly of crystallizable block copolymers can involve multiple levels of structural hierarchy and heterogeneity in the final particles. Therefore, caution should be exercised during the interpretation of SAXS data, and it may require the use of other characterization tools for a quantitative form factor analysis.

**Figure 6:**
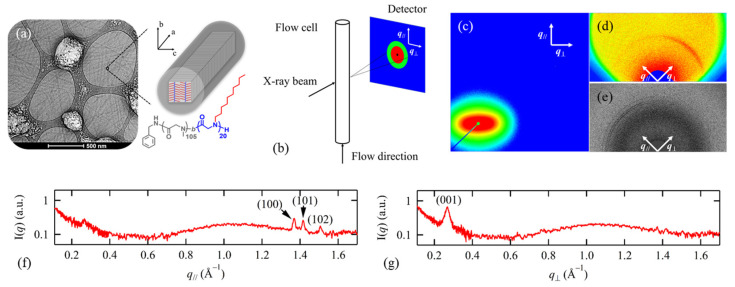
(**a**) Cryo-TEM image for PNMG_105_-*b*-PNDG_20_ in diluted methanol solution; (**b**) schematic experimental setup for a capillary cell under unidirectional flow conducting X-ray; two-dimensional (**c**) SAXS, (**d**) MAXS, and (**e**) WAXS images for PNMG_105_-*b*-PNDG_20_ in methanol solution measured under a unidirectional flow; the corresponding one-dimensional profiles of the MAXS/WAXS profiles along the (**f**) *q*_//_ and (**g**) *q*_┴_ direction. Reprinted from Ref. [29] with permission from the American Chemical Society.

It is known that amphiphilic coil-crystalline diblock copolypeptoids with long alkyl side chains can self-assemble into fiber-like structures in dilute solution through CDSA [29,33,96]. For instance, Lee et al. showed that poly(N-methyl glycine)-*b*-poly(N-decyl glycine) (PNMG-*b*-PNDG), with relatively low volume fraction, can slowly self-assemble into long, worm-like nanofibrils in dilute methanol solution [96]. Using cryo-TEM, the morphological transition from spherical micelles to short rods, and then to long nanofibrils with a uniform diameter, was observed [96]. However, the detailed molecular arrangement of each block within these nanofibrils remains poorly understood. To address this question, Jiang et al. investigated the solution self-assembly of PNMG_105_-*b*-PNDG_20_ in dilute methanol using solution X-ray and neutron scattering. They found that the 1D SANS and SAXS profiles of the nanofibrils could be well-fitted by the scattering model for cylindrical micelles developed by Pedersen and co-workers [108,109], indicating that the elongated nanofibrils comprise a collapsed PNDG core and a shell with swollen PNMG chains. To gain insights into the molecular packing within the collapsed crystalline core, solution X-ray scattering was measured under different flow rates during the unidirectional flow of the nanofibrils solution inside the capillary cell (Figure 6b) [29]. Under static conditions, the 2D scattering patterns of the nanofibrils solution captured by all three detectors (which covers an effective *q*-range of ∼0.0015 to 1.7 Å^−1^) are isotropic in all directions. By contrast, the scattering patterns become anisotropic under a unidirectional flow with a constant shear rate of ~25.6 s^−1^ near the wall (Figure 6c–e), suggesting that the 1D nanofibrils were preferentially aligned parallel to the *q*_//_ direction, i.e., the flow direction. More importantly, the scattering peak due to (001) reflection, which corresponds to the side-by-side packing of PNDG, is more pronounced in the *q*_┴_ direction, whereas the (100) reflection, which corresponds to the face-to-face packing of PNDG, is more intense along *q*_//_ (Figure 6f,g). This suggests that the 1D elongation of PNMG_105_-*b*-PNDG_20_ nanofibrils is induced by the face-to-face packing of PNDG backbones along the crystallographic *a* direction, while the lateral diameter of the nanofibrils is determined by both the backbone length and crystalline packing of PNDG along the *c* direction (Figure 6a). 

**Figure 7:**
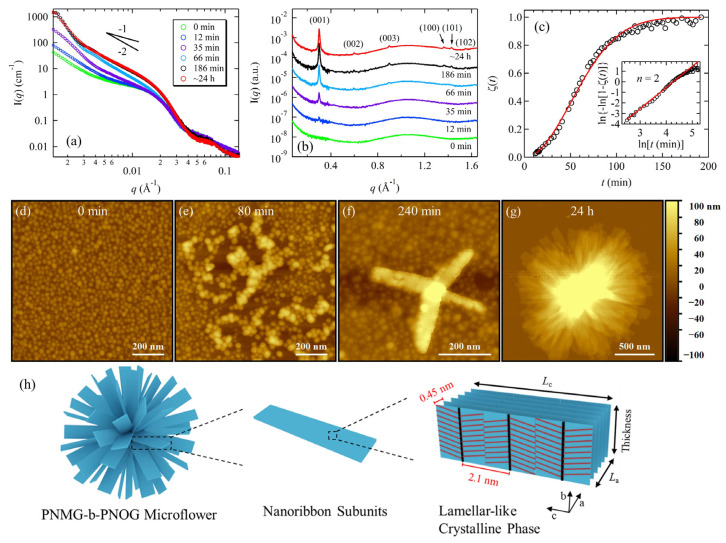
(**a**) SAXS profiles and (**b**) corresponding WAXS profiles of the 5 mg/mL PNMG_116_-*b*-PNOG_94_ methanol solution at different waiting times (*t*): 0 min–24 h; (**c**) ζ(t) values (black circles) obtained from normalized integrated intensity of the (001) peaks at different t (Inset shows the Sharp-Hancock plot, where the red solid lines correspond to the best fits to the data); (**d**–**g**) AFM height images for PNMG_116_-*b*-PNOG_94_ self-assemblies after the diluted methanol solution was cooled to room temperature at different waiting times (*t*): 0 min–24 h; (**h**) schematic illustration of molecular arrangement inside the PNMG_116_-*b*-PNOG_94_ microflower. Reprinted from Ref. [32] with permission from American Chemical Society.

Another advantage of X-ray scattering is that it allows for time-dependent studies of the self-assembly of polypeptoids in their natural solution environment. For dilute solutions, the typical temporal resolution for a synchrotron-based SAXS measurement ranges from less than a second to a few minutes. In a recent study, Kang et al. used synchrotron X-ray solution scattering to study the hierarchical self-assembly of amphiphilic diblock copolypeptoids, namely poly(N-methyl glycine)-*b*-poly(N-octyl glycine) (PNMG-*b*-PNOG) [32]. To induce solution self-assembly, a 5 mg/mL PNMG-*b*-PNOG solution was first heated to a high temperature to ensure the complete dissolution of the polymers in methanol. In situ SAXS measurements confirmed the predominant presence of unimers at high temperatures. Immediately after the solution was cooled to room temperature, the structural evolution of PNMG-*b*-PNOG as a function of time was then monitored by solution SAXS/WAXS, which took several hours to complete. Based on the time-dependent SAXS/WAXS analysis (Figure 7a–c), along with the AFM results (Figure 7d–g), it was found that the overall self-assembly process of PNMG-*b*-PNOG is relatively sluggish and involves the assembly of multi-level building blocks in a stepwise manner. Upon cooling to room temperature, PNMG-*b*-PNOG first associated into amorphous spherical micelles with an average diameter of ~30 nm due to the solvophobic interactions (Figure 7d). These amorphous micelles then further aggregated and re-arranged into nanoribbons (Figure 7e–g) due to the 2D crystalline growth of the PNOG block, as evidenced by the I(*q*)~*q*^−2^ dependence in the SAXS region (Figure 7a) and the Avrami analysis on the time-dependent WAXS data (Figure 7c). Finally, large microflowers composed of stacked nanoribbons were formed, in which the board-like PNOG segments were stacked face-to-face (along the *a* direction) and side-by-side (along the *c* direction) in the crystalline lattice (Figure 7h). The simultaneous evolution in both SAXS and WAXS profiles also revealed a strong correlation between the hierarchical self-assembly of PNMG-*b*-PNOG at the nanometer scale and the crystallization of the PNOG blocks at the molecular level. 

Besides solution X-ray scattering, small-angle neutron scattering (SANS) can also be used to investigate the self-assembly of polypeptoids in solution. Unlike SAXS, SANS utilizes neutrons as probes, which interact with nuclei within matter, making it more sensitive to light elements and isotopes [72]. Isotopic substitution and contrast variation can enhance the neutron-scattering contrast between different components in the sample solution, allowing for the selective probing of a particular component or region of interest within the self-assembled structures. For example, SANS is highly sensitive in determining the detailed structural parameters of polymeric micelles that feature a densely packed core region and swollen outer chains, such as the core-shell structure, sizes, spatial distribution of polymer chains, and degree of solvent penetration [110]. To obtain accurate results, the incoherent scattering contribution from the micellar solution should be minimized, and the appropriate data reduction protocols must be used to extract the true scattering signal from polymer micelles. In some cases, simultaneous SANS and SAXS analysis on the same sample can provide better opportunities for researchers to resolve mathematical problems during modeling and data analysis, leading to a more reliable interpretation of the scattering data in reciprocal space [111,112,113]. However, due to the lower flux density of the neutron beam, which is often several orders of magnitude lower than that of synchrotron X-rays, SANS typically requires longer exposure times to obtain adequate signal-to-noise ratios. As a result, time-dependent SANS studies may have poorer time resolution compared to SAXS, limiting their ability to capture rapid changes in structure during solution self-assembly.

**Figure 8:**
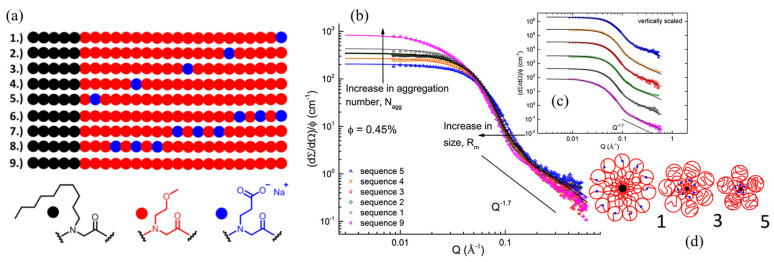
(**a**) The sequences of ionic peptoid block copolymers showing the singly charged series (1–5), triply charged series (6–8), and the charge-neutral sequence (9); SANS scattering intensity, normalized by the polymer volume fraction and analysis of the micellar solution of six sequence-defined polypeptoid block copolymers, bearing single ionic monomer in 0.45 vol% D_2_O (pH9). (**b**) Fitted data for the singly charged peptoid polymer series and (**c**) the data vertically scaled by a constant for clarity; (**d**) cartoon represents the micellar structure of peptoid 1, 3, and 5 regarding to the ionic monomer position along the peptoid chains. Reprinted from Ref. [15] with permission from the American Chemical Society.

Sternhagen et al. [15] studied the impact of the number and position of ionic monomers on micellar structure using a series of polypeptoids comprising a total of 25 monomer units (DP = 25) with three different types of N-substituents: n-decyl (hydrophobic), 2-methoxyethyl (neutral), and 2-carboxyethyl (ionic) (Figure 8a). The monomer sequence in the hydrophilic segment is systematically varied to achieve precise control over the position of ionic monomers along the chain for singly charged and triply charged sequences. The detailed micellar structures of these amphiphilic ionic polypeptoid BCPs in dilute D_2_O solutions, with fixed concentration and pH, were characterized by SANS. The SANS profiles showed a Guinier-like low *q* region and a power law dependence on *q* with a −1.7 exponent at high *q*, indicating that the inter-micellar interactions could be neglected, and that all peptoid micelles had swollen corona chains in the outer region (Figure 8b,c). A core−shell form factor, as previously reported by Richter and coworkers [114], was used to fit the SANS data, which allowed for the determination of several structural parameters of the micelles, such as the micellar aggregation number, size, core radius, corona thickness, core−corona interfacial area per polymer chain, and corona volume occupied per polymer chain. The analysis of the relationship between these structural parameters and the position/total number of ionic monomers along the hydrophilic segment revealed the effect of electrostatic interactions on the micellar structure. As illustrated in Figure 8d, when the ionic monomer position was moved closer to the junction of the hydrophilic and hydrophobic segments, a systematic decrease in the aggregation number and the micellar size was observed due to the enhanced electrostatic repulsion.

## 3. Conclusions and Outlook

In this review, we provide an overview of the recent experimental advances in investigating the self-assembly and phase behavior of polypeptoids. We mainly focus on the use of scattering and microscopy techniques, which enable researchers to unravel multiscale structural features and assembly processes of polypeptoids over a wide range of length scales and time scales. By using these complementary characterization tools, researchers are able to gain a more comprehensive understanding of the relationship between the chemical structure, molecular arrangement, and hierarchical self-assembly behavior of polypeptoids in bulk, thin film, and solution states, paving the way towards the rational design of peptoid-based materials with tailored properties and functionalities. 

Looking to the future, there are several promising avenues for experimental characterization of peptoid-based self-assemblies. One important direction is in the development of advanced in situ characterization techniques that can provide higher spatial/temporal resolution and sensitivity for more in-depth analysis of solution self-assembly of polypeptoids. This would allow for more systematic and quantitative studies on the effects of annealing conditions, temperature, ionic strength, and external forces on the self-assembly of polypeptoids. For solution scattering using X-ray and neutron sources, there is a need to develop more refined analytical models and approaches for the quantitative analysis of many sophisticated hierarchical nanostructures formed by polypeptoids, such as microflowers and superbrushes. In addition, the integration of experimental characterization methods, with theoretical and computer simulation approaches, is expected to offer new opportunities for a deeper understanding of the fundamental principles that govern the self-assembly and phase behavior of polypeptoids. Quantum-mechanical (QM) modeling [115], all-atom molecular dynamics (MD) simulations [74,116,117], coarse-grained (CG) simulations [118,119], and Monte Carlo simulations [120] have shown their capabilities in investigating the self-assembly and dynamical properties of polypeptoids in various states, enabling researchers to better explain and rationalize the experimental evidence observed from scattering and microscopy techniques.

Nevertheless, as countless polypeptoids with different chain lengths, side chain structures, chirality, sequence, and architecture can now be designed and synthesized by chemists in an effective fashion, the high-throughput characterization and rapid screening of these vast libraries of polymeric materials remain challenging. While we have demonstrated that synchrotron-based solution scattering and advanced cryo-TEM are powerful tools for understanding the self-assembly mechanism, they are typically available at a few world-class facilities. As regular access to these facilities is often limited, researchers are still facing significant obstacles in gaining rapid understanding and feedback regarding the peptoid materials they have synthesized. To fulfill the characterization needs of polypeptoid self-assemblies, it would be beneficial to advance the development of more affordable, laboratory-based scattering/microscopy techniques that can provide the necessary information at relevant length and time scales with sufficient sensitivity. The development of software or programs that allow the automated verification, reduction, modeling, and analysis of the data based on machine learning would also be helpful to accelerate the pace of research. We expect that these advancements will enable researchers to fully unlock the full potential of polypeptoids as protein-mimetic materials with exceptional properties and functionalities, significantly enhancing their applications in drug delivery, biosensing, gene therapy, and biomimicry. 

## Data Availability

Not applicable.
